# Restoration Effects of the Riparian Forest on the Intertidal Fish Fauna in an Urban Area of the Amazon River

**DOI:** 10.1155/2016/2810136

**Published:** 2016-09-07

**Authors:** Júlio C. Sá-Oliveira, Stephen F. Ferrari, Huann C. G. Vasconcelos, Raimundo N. G. Mendes-Junior, Andrea S. Araújo, Carlos Eduardo Costa-Campos, Walace S. Nascimento, Victoria J. Isaac

**Affiliations:** ^1^Ichthyology and Limnology Laboratory, Universidade Federal do Amapá-NEPA (UNIFAP-NEPA), Rodovia JK, km 02, 68.903-419 Macapá, AP, Brazil; ^2^Department of Ecology, Universidade Federal de Sergipe (UFS), São Cristóvão, SE, Brazil; ^3^Ichthyology and Limnology Laboratory, Universidade Federal do Amapá (UNIFAP), Rodovia JK, km 02, 68.903-419 Macapá, AP, Brazil; ^4^Cajari River Extractive Reserve, Instituto Chico Mendes de Conservação da Biodiversidade (ICMBio), Rua Leopoldo Machado, 1126 Centro, Macapá, AP, Brazil; ^5^Zoology Laboratory, Universidade Federal do Amapá (UNIFAP), Rodovia JK, km 02, 68.903-419 Macapá, AP, Brazil; ^6^Herpetology Laboratory, Universidade Federal do Amapá (UNIFAP), Rodovia JK, km 02, 68.903-419 Macapá, AP, Brazil; ^7^Fishery Biology Laboratory, ICB, Universidade Federal do Pará (UFPA), Av. Perimetral 2651, Guamá, 66077530 Belém, PA, Brazil

## Abstract

Urbanization causes environmental impacts that threaten the health of aquatic communities and alter their recovery patterns. In this study, we evaluated the diversity of intertidal fish in six areas affected by urbanization (areas with native vegetation, deforested areas, and areas in process of restoration of vegetation) along an urban waterfront in the Amazon River. 20 species were identified, representing 17 genera, 14 families, and 8 orders. The different degrees of habitat degradation had a major effect on the composition of the fish fauna; the two least affected sectors were the only ones in that all 20 species were found. Eight species were recorded in the most degraded areas. The analysis revealed two well-defined groups, coinciding with the sectors in better ecological quality and degraded areas, respectively. The native vegetation has been identified as the crucial factor to the recovery and homeostasis of the studied ecosystem, justifying its legal protection and its use in the restoration and conservation of altered and threatened environments. These results reinforce the importance of maintaining the native vegetation as well as its restoration in order to benefit of the fish populations in intertidal zones impacted by alterations resulting from inadequate urbanization.

## 1. Introduction

The diversity of Amazonian fishes is well documented in general, especially for the communities that inhabit the unique aquatic systems of this vast river basin [1–3]. However, the diversity of the fish communities of the intertidal zones of the estuary of the Amazon River, which are characterized by an enormous complexity of environments over time and space [4, 5], is still relatively poorly understood. This estuary encompasses an enormous area, which includes Marajó Archipelago, and its discharge of freshwater has a major influence on both fluvial and marine ecosystems [6, 7].

Surveys of fish communities are important for the definition of local diversity and provide data for zoogeographic analyses and inferences on the interrelationships among different aquatic ecosystems. Fish communities may be especially useful as indicators of environmental quality for long-term biological monitoring [8–11].

As the interface between aquatic and terrestrial ecosystems, the intertidal zone is a dynamic environment in which the diversity of organisms may exceed that of more homogeneous neighboring systems [5, 12]. Ecologically, this zone represents a rich source of feeding resources and refuges for many different types of organisms, but it is also subject to major environmental fluctuations on both diurnal and annual cycles [13–15]. In an environment with relatively shallow waters, however, there is a tendency for the resident organisms to be of small size, in many cases the juveniles, rather than the adults of a given species, reinforcing the importance of this zone as a breeding ground or nursery area for many organisms [16].

The fish communities of tidal zones are subject to the influence of tidal cycles and annual fluctuations in river levels and, increasingly, in many areas, the effects of anthropogenic impacts [17]. These impacts may result in the loss of specific habitats and a decline in the populations or the local extinction of the populations of some organisms [8]. Tidal pools may be inhabited by permanent or temporary residents, which may be found in these environments for periods ranging from a few days to a number of years, or occasional visitors [12, 16, 18].

Despite the clear ecological importance of the freshwater on estuarine intertidal zone of the Amazon River as a nursery area and feeding ground for many species, few data are available on the ichthyofauna of these environments. The understanding of the dynamics of this unique type of system, based on the analysis of environmental features and biological parameters (species richness and diversity, population density), may also contribute to the comprehension of the effects of environmental impacts.

Among the main degradation instances moved by the urbanization process, the removal of natural vegetation is the most expressive, which usually leads to the creation of isolated fragments immersed in an anthropic matrix [19]. This type of degradation promotes alteration of physical, chemical, and biological parameters of the degraded system, altering the energy availability and flow of organisms [20]. Riparian forests in altered landscapes can be vital to wildlife conservation [21]. In fragmented environments, riparian vegetation behaves as an important biodiversity corridor in promotion necessary for interpatch movement [22], migration [23], and dispersion [24].

Studies have proven substantial reduction of the richness of fish related to alterations caused by urbanization processes, such as the reduction of vegetation and changes in water quality, in addition to domestic and industrial waste, as well as the excessive removal of individuals by fishing [25–28]. In the Amazon, urbanization follows an exaggerated process of deforestation, which threatens all natural biodiversity [29], with the city of Macapá being in these areas, which undergo a wide process of anthropization without appropriate planning.

In this context, the present study evaluated the characteristics of the ichthyofauna of the intertidal zone of the waterfront of the city of Macapá at the mouth of the Amazon River and environmental conditions of the area under study, influenced by the anthropogenic factors. Our hypothesis predicts that the presence of native vegetation in the intertidal zone is one of the factors capable of minimizing the actions of inadequate urbanization in the intertidal zone, such as building of homes and discharge of effluents, waste, and traffic vessels, among others, and that maintaining this vegetation is able to restore preterit diversity to human intervention to alteration of the environment.

## 2. Materials and Methods

### 2.1. Study Area

**Figure 1:**
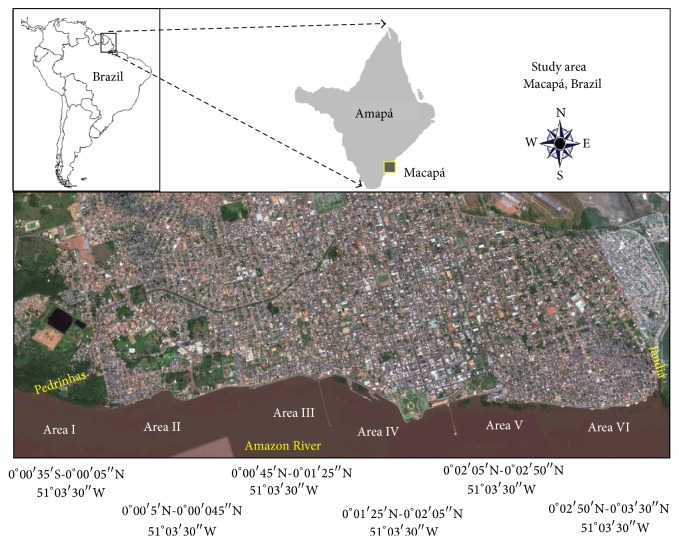
Study area: intertidal zone of the Amazon River on the waterfront of the city of Macapá in Amapá, Brazil. Source: Google Earth.

The present study focused on the intertidal zone of the northern margin of the Amazon estuary (Figure 1), specifically the 3,5 km waterfront of the city of Macapá between Pedrinhas (0°00′35′′S, 51°03′30′′W) and Jandiá streams (0°03′30′′N, 51°03′30′′W). The substrate within this area is clayey-sandy, and the predominant types of vegetation are mangrove forest (*Avicennia germinans*), aquatic macrophytes (*Eichhornia* sp. and* Paspalum* sp.), and typical Amazonian freshwater swamp forest. This area is characterized by a considerable degree of environmental degradation, resulting from human activities, such as landfills, shipping, and the discharge of untreated domestic effluents into the river water and total removal of riparian and aquatic vegetation (Table 1). The annual variation in the level of the Amazon River near its mouth is mediated by the daily tidal cycle, with amplitude of approximately 3.8 m and salinity of 0.0 ppm [30, 31].

### 2.2. Sampling Procedures

Specimens were collected using beach trawls and hand nets of 1 m^2^ each, in February, April, August, and September 2010. The study area on the Macapá waterfront between Pedrinhas and Jandiá streams was divided into six areas: (I) Pedrinhas stream-Atúria; (II) Atúria-Araxá; (III) Santa Inês-Fortaleza waterfront; (IV) Fortaleza Port-Mulheres stream; (V) Perpétuo Socorro waterfront, and (VI) Cidade Nova waterfront-Jandiá stream (Figure 1). Area V, in particular, has native vegetation restoration, due to the advancement of the river in this area. The resident population has established an agreement among the inhabitants not to remove native vegetation, allowing its restoration and preventing the river advancement. This natural recovery started about 18 years ago. The characteristics of each sector are shown in Table 1. A 500 m transect was established within each sector, perpendicular to the margin of the river, for the collection of fish specimens, which was carried out by three investigators in each sector, who surveyed each transect thoroughly at low tide during a period of two hours during the day and two hours during the night on the same day in each month of the study period, evaluating a standardized number of four (4) water puddles (9 ± 0.65 m^2^ approximately each, with shallow depth between 10 and 20 cm) in each area.

The specimens collected during the study were fixed in 10% formaldehyde for identification and collection of biometric data. Taxonomic identification was based on the keys available for Amazonian fishes [32, 33].

Species richness (*S*) was determined by the number of taxa recorded within each study sector. Diversity was evaluated using the Shannon-Wiener index (*H*′), calculated by *H*′ = −∑(*n*_*i*_/*N*) · log⁡(*n*_*i*_/*N*), where *n*_*i*_ is the number of individuals of the *i*th species and *N* is the total number of individuals recorded during the study, and Pielou's equitability index (*E*), *E* = *H*′/log⁡*S*, where *H*′ is the Shannon-Wiener diversity index and *S* represents species richness. The dominance index (*D*) was calculated as in [34], using the equation *D* = ∑*n*_*i*_(*n*_*i*_ − 1)/*N*(*N* − 1), where *S*, *n*, and *N* are defined as above [35].

### 2.3. Statistical Analyses

The variation in the community parameters (species richness, Shannon-Wiener's diversity index, equitability, and dominance) among zones was analyzed using one-way Analysis of Variance (ANOVA). The normality of the data was assessed using the Kolmogorov-Smirnov and Shapiro-Wilk tests, and homocedasticity was assessed by the Levene and Bartlett tests. When these assumptions were not satisfied, the data were square-root-transformed prior to analysis. Significant ANOVA results were analyzed using the Tukey test. When the prerequisites for parametric analysis were not met, even after the transformation of the data, the nonparametric Kruskal-Wallis and Dunn tests were used [36, 37].

Multivariate cluster analyses and nonmetric multidimensional scaling (NMDS) were applied to the data from each sector for the evaluation of the spatial variation in the composition and abundance of the species collected during the study. For this analysis, the species abundance values were square-root-transformed to produce a similarity matrix based on the Bray-Curtis coefficient, with the resulting groups being estimated by the simple mean UPGMA similarity [38]. The SIMPROF procedure was used to evaluate the similarity of the assemblages among areas based on the relative contribution of each species. The analyses were run in PAST 2.09 [39], R version 2.12.2 [40], and BioEstat 5.0 [41], considering *p* = 0.05 significance level for all tests.

A multiple logistic regression model was constructed to verify the qualitative factors associated with variation of the species richness (*S*) in each area. In this analysis, binary logistic regression was used with richness (*S*) as the dependent variable (*Y*), with independent predictive variables being the qualitative environmental characteristics of the surroundings of each area: (*X*1) presence/absence of vegetation, (*X*2) presence/absence of effluents, and (*X*3) presence/absence of residences. High species richness was coded as 1 and low richness as 0. The presence of vegetation was coded as 1 and the presence of effluents and residences as 0. The general equation of the logistic model was Logit Pi (*S*) = *α* + (*βX*1 vegetation) − (*βX*2 effluents) + (*βX*3 residences).

The method used to select the logistic model was the Enter. To evaluate the accuracy of the predictive power of the model, through evaluation of the dependent variable and not the likelihood criterion, the Hosmer-Lemeshow test was used [42], considering *α* = 0.05. The analyses were run in BioEstat 5.0 [41].

## 3. Results

### 3.1. Composition of the Ichthyofauna

The fish fauna of the study area includes a total of 20 species belonging to 17 genera, 14 families, and 8 different orders (Table 2), based on the 767 specimens collected during the study period. Four of the families (Loricariidae, Pimelodidae, Sciaenidae, and Clupeidae) were relatively abundant, with more than 10% of the specimens collected (Figure 3). The most abundant species were* Rhinosardinia amazonica* and* Vandellia* sp.

Two orders (Siluriformes and Characiformes) accounted for 60% of total species richness, while the other orders were represented by no more than one or two species. Two families, Clupeidae (*n* = 154 individuals) and Trichomycteridae (*n* = 104), accounted for more than one-third of the specimens collected, while Heptapteridae, Achiridae, and Erythrinidae were the least abundant (Table 2).

**Figure 2:**
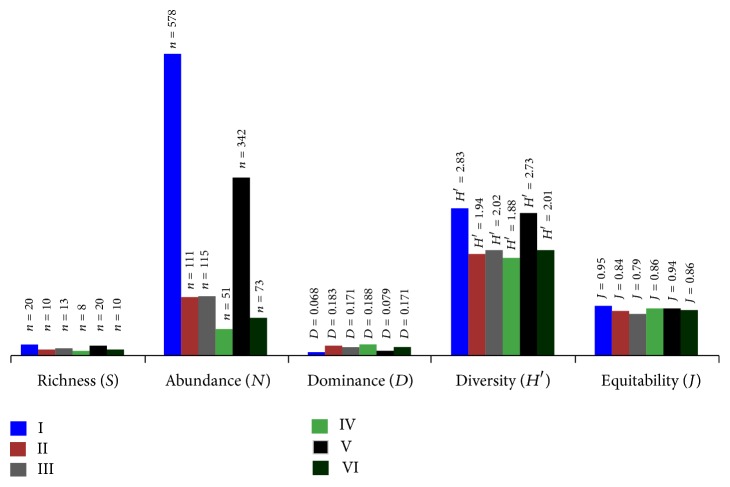
Ecological indices of the ichthyofauna from intertidal zone of the Macapá waterfront in northern Brazil.

*Astyanax bimaculatus*,* Hypostomus plecostomus*,* Hypostomus emarginatus*,* Vandellia* sp., and* Hoplosternum littorale* were the only species captured in all six sectors (Table 3), while sectors I and V were the only ones in which all the species were recorded. In some cases (*Colomesus psittacus*,* Achirus achirus*, and* Potamorrhaphis guianensis*), the species were only recorded in these two sectors. These two sectors were also responsible for the highest levels of relative abundance of most of the species recorded in the study and the largest numbers of individuals recorded in general (Figure 2 and Table 3).

In terms of ecological indexes, area IV had a much smaller number of species, reaching only eight species, which also had the lowest abundance, with less than 10% of the number of specimens collected in comparison with sector I (Figure 2 and Table 3). There were significant differences for richness, diversity, dominance, and equitability between areas, with areas I and V being equal to one another, but different from others, with sectors I and V being characterized by high diversity and equitability and low species dominance in comparison with the other sectors (Table 3).

**Figure 3:**
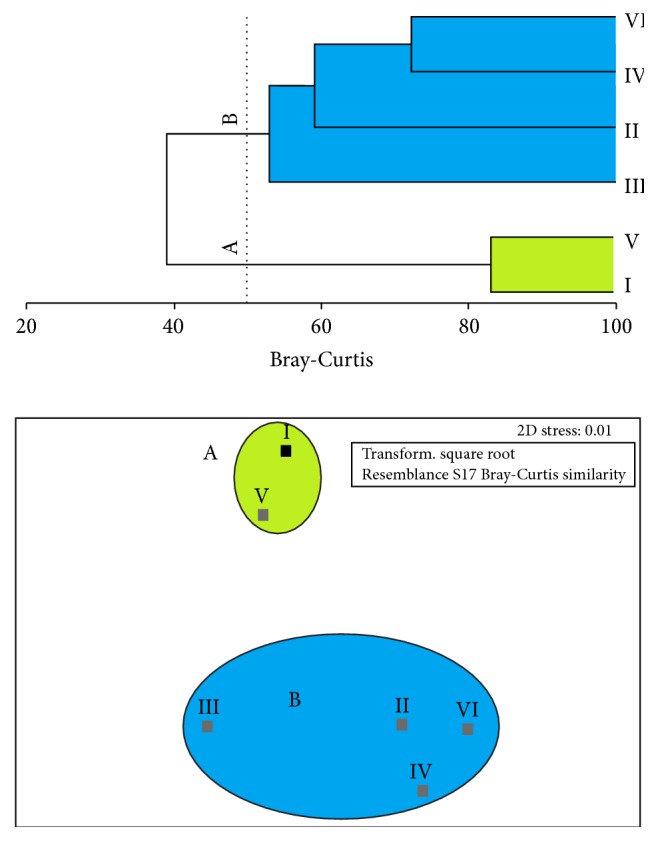
Cluster analysis and nonmetric multidimensional scaling (MDS) analysis of the study areas, indicating two clear groups: A, containing only areas I and V, and B, encompassing all others area (II, III, IV, and VI).

The cluster and ordination analyses (Figure 3) identified two distinct groups, with similarity of over 50%. The first (group A) includes the two sectors with high species richness, diversity, and equitability, while the second (group B) encompasses the remaining sectors. The species that most contributed to the definition of group A were* Hoplerythrinus unitaeniatus*,* Triportheus angulatus, Pimelodus blochii*,* Achirus achirus*, and* Potamorrhaphis guianensis*. In the case of group B, the most relevant species were* Vandellia* sp.,* Anableps anableps*,* Plagioscion squamosissimus*, and* Hoplosternum littorale.*

The logistic model was defined by the following equation: logit (richness (*S*)) = −1630 (constant) + 3.3181 (vegetation) − 0794 (effluents) + 2.7620 (residences). The indicators demonstrated that the values of the variables “vegetation” and “residences” positively influence the construction of the model. In another way, the variable “effluent” presents a negative value, indicating that, with lower values of this index, there will be greater probability for elevated richness.

The overall statistics were significant (score = 11,20; df = 1; *p* = 0.01), as well as the score values of the vegetation (score = 7.889; df = 1; *p* = 0.005), followed by residence (score = 6.454; df = 1; *p* = 0.01), indicating that the coefficients for the model variables are significantly different from zero.

The values of Exp (*B*) (odds ratio) indicate that the species richness is about 27 times more likely to be elevated if area native vegetation is displayed, such as trees and shrubs, and 15 times more likely if there are no residences in the surroundings. The variable vegetation alone explains 41.66% of the variation in the areas of richness; however, when associated with the variable effluent (absence), the probability of the presence of vegetation influencing the richness increases to 83.62%. Finally, when the variable vegetation is associated with the absence of residences, the probability values rise to 91.86%. The presence of effluents affects only 2.52% in the variation of the species richness (Table 4).

The estimated coefficients by means of the model showed satisfactory adjustment, with *R* ^2^ Nagelkerke (“pseudo-*R* ^2^”), resulting in 0.689; that is, 68.9% represents the proportion of the variance of the dependent variable, which is explained by the independent variables. The significance level as measured by the Hosmer and Lemeshow test was 43%, that is, more than 5%; so it can be affirmed that there is an association between predicted and real values.

## 4. Discussion

Approximately 25,000 species of fish are recognized worldwide, of which 40% are found in freshwater environments [43]. The ichthyofauna of the Neotropics is characterized by its high species diversity, with an estimated five to six thousand species in all [42, 44]. The majority of this diversity is found in the Amazon basin [1], although it seems likely that the full diversity of this fauna has yet to be described [44].

A predominance of Characiformes and Siluriformes and, to a lesser extent, Perciforms was recorded in the present study. These orders dominate the fluvial systems of South America [1, 45, 46]. Loricariidae, the most diverse Siluriformes family [46], and Pimelodidae were the most species-rich families, although trichomycterids were more abundant, overall. This reflects more general patterns of diversity found in the Amazon basin as a whole [1].

Species richness is a basic and universal parameter for the understanding of the diversity of an ecosystem [35], and the total of 20 fish species recorded in the present study is relatively low by the standards of the Amazon basin and in particular the main Amazon channel. However, as the present study focused on the fish fauna of the intertidal zone at low tide, it was undoubtedly biased towards the groups of fishes best adapted to the unique hydrological conditions found in this environment. While the fish fauna of this intertidal zone may be naturally poor in species in comparison with other zones of the estuary, due to the specific characteristics of this environment, this scenario may also be related to the effects of anthropogenic impacts. Despite these impacts, the ecological diversity of the different sectors of the study area (*H*′ = 1.88–2.82) was within the range recorded for aquatic systems in the Amazon basin, where indices vary from 0.82 to 5.44 [47].

The populations of the study area were relatively well balanced, in general, although sectors I and V were the most homogeneous in terms of the numbers of individuals. The abundance of fishes within the study area was related primarily to the dominance of* Rhinosardinia amazonica* (14.8% of the individuals captured) and* Vandellia* sp. (13.5%). These values are very similar to those recorded for the dominant fish species in assemblages of other aquatic systems in the Amazon basin [47].

The cluster and similarity analyses defined two principal groups: the first (A) formed by the assemblages present in sectors I and V and the other (B) encompassing the remaining sectors (II, III, IV, and VI). The two sectors in group A were clearly the least impacted of the environments, with the lowest discharge of effluents and the amplest cover of native vegetation, and presented the highest species richness, diversity, and equitability. It seems likely that the more natural conditions found in sectors I and V are more favorable to the maintenance of community structure [47]. However, the reduced occurrence and density of individuals in the other sectors (group B) may be related to local impacts, such as the reduction or loss of the riverbank vegetation, eutrophication, pollution, and silting, which may have direct effects on dissolved oxygen concentrations and other limnological parameters, in particular total nitrogen and phosphorus concentrations [48]. The predominance of ecologically more tolerant species over the more sensitive ones reinforces the role of these disturbances in community structure, as found in this study and in other fish communities [49]. The fact that areas I and V are not contiguous seems to support this hypothesis.

While the waterfront of Macapá city is relatively homogeneous, varying levels of anthropogenic impact have fragmented the local intertidal zone into distinct sectors, reflecting different levels of human intervention and native vegetation cover. These sectors are reflected in the differential distribution of fish species across the intertidal zone, with a clear relationship between species richness and anthropogenic impact, in particular with regard to vegetation cover, evidenced in the logistic regression analysis. The different levels suggest a potential role of these least impacted areas as refuges that may aid the partial recovery of the fish fauna in adjacent more degraded areas.

Environmental changes of anthropogenic origin are reflected in limnological conditions of the environment and in food and reproduction of the species [50–52]. In these cases, the composition and structure of communities, such as fish, suffer interference, being mainly composed of opportunistic species (r-strategists) [53], as* Hypostomus plecostomus*,* Hypostomus emarginatus*,* Colomesus psittacus*,* Hoplosternum littorale*, and* Vandellia* sp., the latter attracted by human excreta, such as ammonia [54]. However, when the change is mitigated, as in the riparian forest restoration, original community recomposition occurs before the disturbance, with an increase in the richness and diversity of species, a fact observed in area V of this study.

The central hypothesis of this study was therefore supported, indicating that the maintenance of native vegetation serves as a buffer to preserve species richness in the face of urban development. It is clear, therefore, that native vegetation represents an important factor in the rehabilitation of Amazonian intertidal ecosystems, justifying its legal protection for the restoration and conservation of aquatic environments.

Based on these results, we believe that additional research on a wide selection of intertidal biota is needed in order to track fluctuations in species composition and abundance and to determine how these communities respond to the enormous environmental changes resulting from the urbanization process, by also quantifying the degree of disruption through physical-chemical analysis of water and sediment. Such information will advance our understanding of the anthropogenic impacts upon the biota of Amazonian intertidal communities and contribute to the development of environmental restoration actions.

## Figures and Tables

**Table 1:** Characteristics of the study sectors surveyed on the waterfront of Macapá, in the Brazilian state of Amapá.

Variable	Area					
	I	II	III	IV	V	VI
Geographic coordinates	0°00′35′S–0°00′05′′N	0°00′5′N–0°00′045′′N	0°00′45′N–0°01′25′′N	0°01′25′N–0°02′05′′N	0°02′05′N–0°02′50′′N	0°02′50′N–0°03′30′′N
Vegetation	Area in a better environmental condition with native forest, with trees, shrubs, and aquatic macrophytes abundant	Open areas mostly with no vegetation, but occasional herbaceous vegetation	Open areas mostly with no vegetation, but occasional herbaceous plants	Mostly with no vegetation, but with occasional aquatic macrophytes	Vegetation in natural restoration process, with trees, shrubs, and aquatic macrophytes	Open area with no vegetation
Impacts	Stilt housing; discharge of domestic effluents	Stilt housing; bars; discharge of domestic effluents; polluted drains	Residential and commercial buildings; bars; port installations, discharge of domestic and industrial effluents; large quantities of rubbish; polluted storm drains	Leisure area; commercial buildings (open air market and shops); bars; residential and commercial effluents; polluted storm drains; port installations; rubbish	Residential and commercial buildings; leisure areas; bars; residential and commercial effluents; polluted storm drains; traffic vessels	Residential buildings; leisure areas; bars; residential and commercial effluents; polluted storm drains

**Table 2:** Occurrence and relative abundance of fish species recorded in the intertidal zone of an urban waterfront on the Amazon.

Order/family	Species	Relative abundance (%) area						
		*n*	I	II	III	IV	V	VI
*Characiformes *								
Erythrinidae	*Hoplerythrinus unitaeniatus* (Agassiz, 1829)	40	52.4	—	—	—	42.9	4.8
Characidae								
Triportheinae	*Triportheus angulatus* (Spix & Agassiz, 1829)	20	63.6	—	—	—	27.3	9.1
Tetragonopterinae	*Astyanax bimaculatus* (Linnaeus, 1758)	88	38.8	14.3	4.1	10.0	20.4	12.0
Serrasalminae	*Serrasalmus marginatus* (Cuvier, 1819)	23	50.0	13.6	—	—	36.4	—
Siluriformes								
Loricariidae	*Hypostomus plecostomus* (Linnaeus, 1758)	68	48.2	10.7	3.6	3.6	28.6	5.4
	*Hypostomus emarginatus* (Valenciennes, 1840)	90	51.2	12.2	2.4	4.9	22	7.3
					—	7.7	23.1	15.0
Auchenipteridae	*Pseudauchenipterus* sp. (Linnaeus, 1766)	60	43.6	10.3	—	7.7	23.1	15.0
Trichomycteridae	*Vandellia* sp. (Valenciennes, 1846r)	184	15.4	26.0	13.0	11.0	20.2	14.0
Pimelodidae	*Pimelodus pictus* (Steindachner, 1876)	32	52.4	—	4.8	—	42.9	—
	*Pimelodus blochii* (Valenciennes, 1840)	29	50.0	—	—	—	44.4	5.6
Heptapteridae	*Pimelodella cristata* (Müller & Troschel, 1849)	15	66.7	—	17.0	—	16.7	—
Callichthyidae	*Hoplosternum littorale* (Hancock, 1828)	84	39.6	11.3	19.0	1.9	22.6	5.7
Cyprinodontiformes								
Anablepidae	*Anableps anableps* (Linnaeus, 1758)	83	42.6	17.0	19.0	4.3	17	—
Perciformes								
Sciaenidae	*Pachyurus schomburgkii* (Günther, 1860)	9	62.5	12.5	13.0	—	12.5	—
	*Plagioscion squamosissimus* (Heckel, 1840)	43	38.5	—	12.0	15.0	23.1	12.0
Tetraodontiformes								
Tetraodontidae	*Colomesus psittacus* (Bloch & Schneider, 1801)	63	71.9	—	—	—	28.1	—
Clupeiformes								
Clupeidae	*Rhinosardinia amazonica* (Steindachner, 1879)	172	48.7	—	16.0	—	35.4	—
	*Rhinosardinia serrata* (Eigenmann, 1912)	80	56.1	—	4.9	—	39	—
Pleuronectiformes								
Achiridae	*Achirus achirus* (Linnaeus, 1758)	39	68.4	—	—	—	31.6	—
Beloniformes								
Belonidae	*Potamorrhaphis guianensis* (Jardine, 1843)	44	87.9	—	—	—	12.1	—

**Table 3:** Mean ± standard deviation of diversity parameters of fish species and abundance recorded in the intertidal zone of the Amazon River at Macapá, Amapá (Brazil).

Index	Areas						*F*_5,18_	*p*	Tukey
	I	II	III	IV	V	VI			
Number of species (*S*)	18.25 ± 6.096	7.25 ± 1.5	7.50 ± 3.69	3.50 ± 2.886	15.75 ± 3.304	6.00 ± 4.082	15.13	*∗∗∗*	a, b, b, b, a, b
Abundance (*N*)	144.5 ±78.66	27.75 ± 14.17	28.75 ± 24.28	12.75 ± 11.35	85.50 ± 15.94	18.25 ± 15.94	6.41	*∗∗∗*	a, b, b, b, ab, b
Dominance (*D*)	0.07 ± 0.006	0.20 ± 0.041	0.20 ± 0.039	0.62 ± 0.437	0.09 ± 0.007	0.36 ± 0.25	17.64	*∗∗∗*	b, ab, ab, a, b, a
Shannon-Wiener (*H*′)	2.72 ± 0.091	1.74 ± 0.162	1.71 ± 0.315	0.78 ± 0.091	2.54 ± 0.130	1.38 ± 0.79	7.83	*∗∗∗*	a, ab, ab, b, a, ab
Equitability (*J*)	0.94 ± 0.005	0.25 ± 0.05	0.50 ± 0.069	0.44 ± 0.050	0.93 ± 0.028	0.55 ± 0.092	15.13	*∗∗∗*	b, ab, ab, a, b, a

^*∗∗∗*^ *p* < 0.001; df = 5 for all indexes. Equal letters together (aa or bb) correspond to no significant differences between areas; different letters separated (a, b) correspond to significant differences between areas; different letters together (ab) correspond to no significant differences between area.

**Table 4:** Parameter estimates, standard errors, Wald test, degrees of freedom, and descriptive level for the final logistic regression model adjusted between the species richness and environmental variables (vegetation, effluents, and residences) on the intertidal zone of the Amazon River at Macapá, Amapá (Brazil).

Variable	*B*	SE	Wald	df	Sig.	Exp (*B*)
Vegetation	3.318	1.301	4.294	1	0.038	27.607
Effluents	−0.794	1.515	0.275	1	0.600	0.452
Residences	2.762	1.280	3.484	1	0.049	15.831
Constant	−1.630	1.172	1.934	1	0.164	5.100

*B*: “*b*” estimates of the parameters of equations; SE: standard error; Wald: Wald statistic; df: degrees of freedom; Sig: significance of the Wald statistics; Exp (*B*): *odds ratio*.
